# Internal anal sphincter augmentation and substitution

**DOI:** 10.1093/gastro/gou004

**Published:** 2014-02-17

**Authors:** Fernando de la Portilla

**Affiliations:** Unidad de Gestión Clínica de Cirugía General y del Aparato Digestivo, Instituto de Biomedicina de Sevilla (IBIS), Seville, Spain

**Keywords:** faecal incontinence, internal anal sphincter dysfunction, internal anal sphincter augmentation, bulking agents

## Abstract

There is an increasing recognition of the importance of internal anal sphincter (IAS) dysfunction presenting as passive faecal incontinence. This problem may manifest after anal sphincterotomy or following the more minimally invasive operations for haemorrhoids, as well as with advancing age. Because of the poor results of IAS plication and the beneficial outcomes with peri-urethral bulking agents in urology, these materials have been developed for use in IAS dysfunction. This review outlines the basic purported mechanisms of action, defining the materials in clinical use, their methods of deployment, complications and reported outcomes. There is still much that is unknown concerning the ideal agent or the volume and the technique of deployment, which will only be answered by powerful, prospective, randomized, controlled trials. The specific role of autologous stem cells designed to regenerate the sphincters in cases of functional impairment or muscle loss is yet to be seen.

## INTRODUCTION

Faecal incontinence affects an estimated 2% of the population. Its prevalence rises with age, with 11% of men and 26% of women over 50 years exhibiting the syndrome [1, 2]. Most patients can be managed conservatively, or by surgical repair if there is a physical disruption of the anal sphincter, with variable results reported because of the multifactorial basis of this disorder. The internal anal sphincter (IAS) provides the greater contribution to resting anal pressure but the vascular tissue of the anal mucosa and submucosa (the anal cushions) may also be important by facilitating hermetic closure of the anal canal [3]. A weak or disrupted IAS or damage to the anal mucosa or submucosa may lead to passive faecal incontinence where incontinence episodes occur without patient awareness. Passive faecal incontinence is the involuntary loss of faeces without the urge to defecate and is predominantly associated with IAS dysfunction.

Incontinence secondary to IAS injury or degeneration has become increasingly recognized and has been more readily documented through the more routine use of endo-anal sonography. The causes of IAS dysfunction can be classified into two main groups: one type is a morphologically intact but functionally weak IAS (a phenomenon which may occur with age as part of sclerotic degeneration or which may result from radiotherapy) [4, 5] and the second type is a structurally damaged IAS, which may occur following anal dilatation or ano-rectal surgery, particularly where the IAS has been deliberately divided for a sphincterotomy in the treatment of chronic anal fissure [6]. Some patients with principally IAS damage can be assisted with anti-diarrhoeal medications including loperamide and codeine, as well as by biofeedback therapies, although the benefits of the latter group of techniques (described elsewhere in this Special Edition) may only be temporary. Other possible but more complex treatments include surgical plication, radiofrequency energy, sacral nerve stimulation and percutaneous posterior tibial nerve stimulation [7–11]. Experience with these techniques for primarily IAS dysfunction is currently limited.

**Figure 1.**
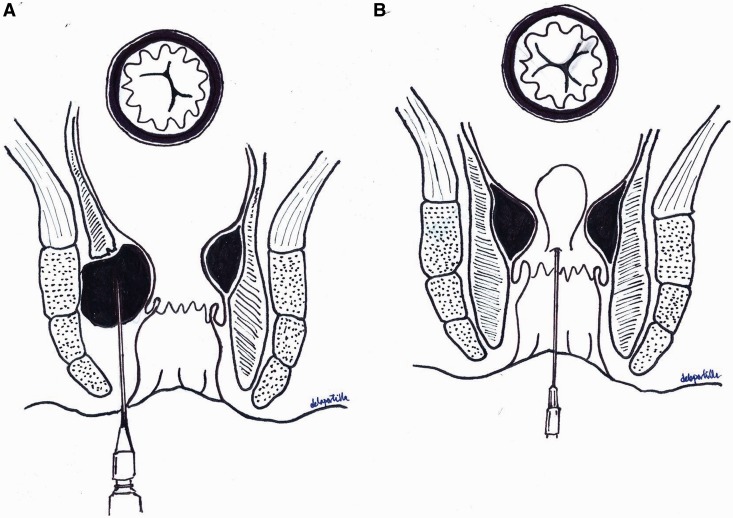
Mechanism of action of bulking agents. A) Injection into an internal anal sphincter defect. B) Injection by quadrants.

The relatively successful application by urologists of injected bulking agents, designed to improve bladder neck closure, has provided an opportunity for their use in the treatment of faecal incontinence due to IAS dysfunction [12]. The mechanism by which bulking agents work remains uncertain (Figure 1). It seems likely that it is purely a mechanical effect of either filling a gutter deformity or raising cushions to keep the anal canal closed and to prevent faecal leakage. Alternatively, they may provide an adequate anal lining that is bulky enough to plug the anal orifice whilst the IAS tonically contracts; however, no reported studies have shown any significant demonstrable effect on the resting or ‘squeeze’ ano-rectal manometry with bulking agents. This article reviews the agents used, the indications for their use, the techniques of deployment, complications and outcomes for IAS bio-augmentation.

## INJECTABLE AGENTS

In ideal terms, a filling material should be non-compatible and non-immunogenic and it should induce a minimal inflammatory and fibrotic response [13]. The agent particles should be large enough to avoid migration away from the injection site (i.e. a diameter >80 μm) and they should be sufficiently durable. Animal studies have shown that there is distant migration of particles with diameters of 4–80 μm, with particulate material found in lymph nodes, the lungs, the kidneys, the spleen and the brain [14]. With migration comes poor durability and, more seriously, the possibility of chronic granuloma formation at the migration site. In general, most of the current materials consist of particles suspended in an excipient, which is usually in the form of a biodegradable gel. The characteristics of an ideal bulking agent are shown in Table 1. Moreover, the carcinogenic potential of implanted prosthetic materials has been examined in animals but it has yet to be established in humans [15, 16].

**Table 1.** Characteristics of the ideal bulking agent

• Biocompatible • Non-migratory • Non-allergenic • Non-immunogenic • Non-carcinogenic • Easy to inject • Produces durable results	

### Polytetrafluorethylene

Polytetrafluoroethylene (Polytef® or Teflon®; DuPont, Wilmington, Delaware, USA) is produced by the pyrolysis of Teflon. Polytef particles range in size from 4–100 μm, with 90% in the 4–40 μm range. The filling material used in the injection treatment is a paste consisting of polytetrafluoroethylene, glycerin and polysorbide. [17].

### Autologous fat

These cells are extracted from the fat of the abdominal wall by suction. They are subsequently purified and suspended in a saline solution and are injected into the anal canal. The rapid digestion and migration potential of this material has stopped the additional development of this option [18].

### Silicone particles

Silicone is the volume augmenter most widely used so far in the treatment of faecal incontinence. The PTQ® implant product is a heterogeneous injectable material consisting of polydimethylsiloxane particles suspended in a bio-excretable carrier hydrogel of polyvinylpyrrolidone (povidone, PVP). The solid particle content represents approximately one-third of the volume and the particle size generally falls within the 100–450 μm range, but there are smaller particles within the gel [19]. Particle flexibility and texture enable collagen deposition in an irregular way around and through the implant.

### Bovine collagen treated with glutaraldehyde (GAX-Collagen or glutaraldehyde cross-linked collagen)

Bovine collagen treated with glutaraldehyde (Contigen®; Bard, Covington, GA, USA) is formed from dermal bovine collagen cross-linked with glutaraldehyde and dispersed in a physiological saline solution saturated with phosphate [20]. The GAX-Collagen contains at least 95% of collagen Type I and between 1% and 5% of collagen Type III [19].

### Carbon beads

This bulking agent (Durasphere®, Carbon Medical Technologies Inc., St. Paul, MN, USA) consists of solid, pyrolytic carbon-coated beads suspended in a viscous carrier gel of water and beta-glucan. The carbon-coated beads are approximately three times the original size and the migration threshold of 80 μm and cannot be absorbed [21].

### Hydroxyapatite ceramic microspheres

This bulking agent (Coaptite®, Bioform, Franksville, WI, USA) consists of hydroxyapatite ceramic microspheres suspended in a carrier gel of sodium carboxymethylcellulose, glycerin and water. The particles are manufactured to be 75–125 μm in size to avoid migration [22].

### Dextranomer/hyaluronic acid co-polymer

This volume augmenter (Solesta™, QMED, Uppsala, Sweden) consists of dextranomer microbeads and non-animal stabilized hyaluronic acid gel (NASHA) with a diameter of 120 μm [23]. The dextranomer facilitates the in-growth of fibroblasts and the production of collagen between the microspheres as the hyaluronic acid is degraded. The bolus is consolidated with endogenous tissue, stabilizing its volume for a sustained, long-term response.

### Cross-linked porcine dermal collagen

This is a biological material (Permacol™, Tissue Science Laboratories plc, Aldershot, UK) containing large particles of cross-linked porcine dermal collagen [24].

### Cross-linked polyacrylamide

This agent is a synthetic, non-particulate hydrogel (Bulkamid™, Contura International A/S, Søeborg, Denmark) consisting of 97.5% water and 2.5% cross-linked polyacrylamide. It is biocompatible but not biodegradable. It is non-resorbable, resistant to migration and known to cause little reaction in the surrounding tissues [25].

### Microprothesis

The Gatekeeper™ (Correggio, Italy) prosthesis comprises a thin, solid polyacrylonitrile cylinder that becomes thicker, shorter and softer within 24 h after implantation, expanding its volume some 720% [26].

### Stem cells

In recent years, several studies have investigated the ability of mesenchymal stem cells (MSC) to differentiate into mature cells of many tissues, both *in vitro* and *in vivo* and to specifically improve tissue repair. These cells have been described for use in different organ systems designed to alter cellular injury. In muscular tissue, in particular, injected MSCs have the capacity to engraft and form multinucleated myotubes, participating effectively in regeneration after injury [27].

## INDICATIONS AND CONTRAINDICATIONS IN TREATMENT WITH BULKING AGENTS

**Figure 2.**
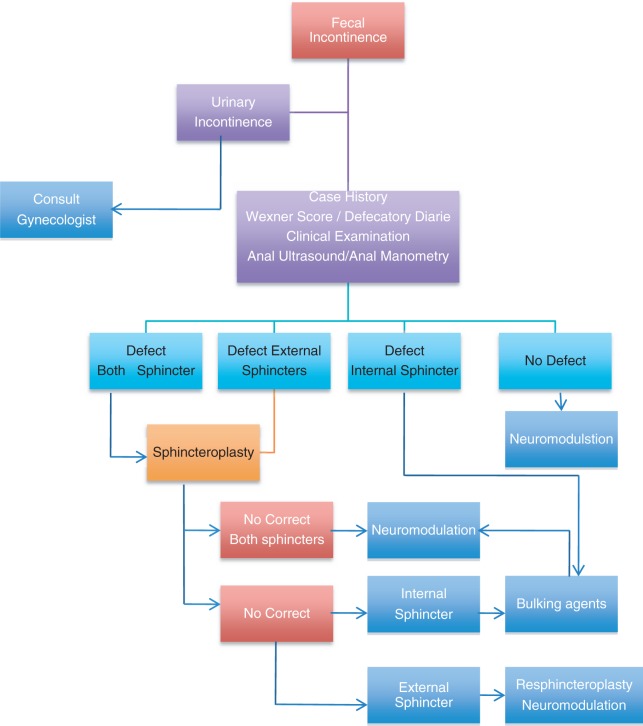
Suggested algorithm for the use of bulking agents in passive faecal incontinence with internal anal sphincter dysfunction.

A suggested algorithm (Figure 2) is shown for managing patients with IAS dysfunction and passive incontinence.

### Indications

- Passive faecal incontinence to solid or liquid stool occurring once per week or more, due to internal anal sphincter dysfunction or related to simple or multiple internal anal sphincter defects, with failure of conservative treatments.

### Contraindications

#### Rectal disorders

- Flatus incontinence only
- External anal sphincter defect
- Significant full-thickness rectal prolapse or mucosal prolapse
- Active ano-rectal sepsis
- Current ano-rectal tumours
- Current anal fissures
- Rectal anastomosis at a level <10 cm from the anal margin
- Active proctitis
- Idiopathic ano-rectal bleeding, rectal varices or vascular malformation
- Ano-rectal stenosis
- Significant chronic ano-rectal or pelvic pain
- Haemorrhoidal disease grade III–IV
- Ano-rectal malformation

#### Concurrent disease/concomitant medications

- Inflammatory bowel disease (IBD)
- Chronic diarrhoea unresponsive to medical treatment
- Medical history of human immunodeficiency virus infection (HIV) or any serious immunocompromised state or administration of immunosuppressive therapy
- Haemorrhagic diathesis or current anticoagulant therapy, such as warfarin, heparin or substances similar to heparin

#### General

- Pregnant women or during the first six months *post partum*
- Age younger than 18 years or older than 80 years

## GENERAL PRACTICAL ASPECTS IN TREATMENT WITH BULKING AGENTS

### Injection preparation

There are several controversies regarding the specifics of injection, which require further clinical study. In general, regardless of the injection site (intersphincteric or submucosal), all bulking agents need only the preparation of the rectum with a simple phosphate enema at least two hours prior to the procedure. Although some protocols suggest that patients use laxative injections at least two days before the procedure in order to soften the stools, we do not consider this to be important for the success of the treatment. There is agreement on the necessity to administer antibiotic prophylaxis before the procedure [28, 29].

### Injection technique

The different agents can be injected either peri-anally, (going through the muscle complex), through the internal anal sphincter, or submucosally. The latter technique is thought to carry a higher risk of erosion and sepsis [30]. The implant can be placed in the intersphincteric plane or in the submucosal plane, always above the dentate line to avoid post-procedural pain. Actually, seven different injection alternatives exist: trans-sphincteric route into the IAS, intersphincteric route into the IAS, intersphincteric route into the submucosa, transanal injection into the submucosa (similar to injection sclerotherapy for haemorrhoids), transsphincteric route into the intersphincteric space, the intersphincteric route into the intersphincteric space and the transsphincteric route injecting submucosally. The injection can be guided digitally (through direct visualization) or by endo-anal ultrasound, where it recently has been suggested that endo-sonographic guidance is associated with improved short-term continence [31], despite previous concerns that its use would disperse the implant. Implants can be injected in four or fewer quadrants, or only in the internal anal sphincter defect site (as after a sphincterotomy) in order to restore the symmetry of the anal canal. The procedure is usually performed in an outpatient setting and can be conducted with local anaesthesia with sedation. Some patients do not require sedation so that, in selected cases, implantation may be conducted as an office procedure. The volume of the injection depends upon the agent employed and the injection method chosen. There is no study comparing the number and location of the implants with the effectiveness of the treatment.

### Post-operative measures

After the procedure, patients should complete a 7–10 day antibiotic course [28, 29]. Some protocols also suggest that patients use laxatives in order to soften the stools, so as to avoid implant compression. Mild analgesics are also suggested for routine use.

### Outcomes

In general, poor outcomes have been reported in the vast majority of available studies but they are usually of a minor nature, consisting of discomfort, pain, bleeding and leakage of injected material [23]. A recently published systematic analysis assessing the effectiveness of volume augmentation showed a shortage of well-designed, randomized trials, with most having methodological weaknesses [23]. Of the five trials analysed, only two have compared the efficacy of bulking agents against placebo. Dextranomer in stabilized hyaluronic acid is the most recent agent to be studied. It has been seen to reduce the number of faecal incontinence episodes by more than 50% in over half of all patients studied [32, 33]. Ratto *et al.*, have recently reported results with the Gatekeeper™ agent in 14 cases, where four prostheses were implanted in the intersphincteric space in each patient under endo-anal ultrasound guidance with local anaesthesia. The mean follow-up was 33.5 months with no complications. There was a significant decrease in major faecal incontinence episodes, along with concomitant reductions in the Cleveland Clinic and Vaizey scores with respect to the pre-treatment baseline [26]. In this study, soiling and the ability to postpone defecation improved significantly and patients reported improvement in their health status and quality of life. On follow-up, manometric parameters had not changed with endo-anal ultrasound, demonstrating that there had been no prosthetic migration. The authors concluded that the Gatekeeper™ anal implant seemed safe, reliable and effective with an encouraging functional improvement in the médium term. In a further recent study by Morris *et al.* comparing Durasphere with PTQ™ implants, there did not appear to be any clinical difference between materials over a 12 month period [34].

In terms of durability, few studies have reported long-term outcomes [31, 35]. Most studies seem to suggest that the benefits of bulking agents appear to dissipate within 6–12 months, when patients may be similarly re-treated; however, loss of treatment efficacy is not generally atrributed to migration [30], but rather to dissipation of the material [36]. In summary, there is clear advantage in the use of bulking agents for primary IAS dysfunction. There are, however, still many aspects of this treatment which require further study. There is no ideal agent and the optimal volume and means of injection are unknown, as are the candidates most likely to benefit from this therapy. Future multi-institutional, randomized studies with rigorous prolonged follow-up are required in order to answer these technical questions. Further research will also define the place of regenerative medicine—implanting autologous fibroblasts which are designed to restore the sphincter muscle fibres.

**Conflict of interest:** none declared.
